# Social Participation Across Midlife and Later Life: Evidence From the Silent Generation

**DOI:** 10.1093/geroni/igaa057.1313

**Published:** 2020-12-16

**Authors:** Eric Vogelsang

**Affiliations:** Cal State University-San Bernardino, San Bernardino, California, United States

## Abstract

Despite the well-established benefits of social participation for individuals and communities, little is known about how it varies throughout the life course. Drawing upon data collected between 1957 and 2011 by the Wisconsin Longitudinal Study (22,023 observations from a cohort of 6,627), this study provides four valuable results. One, I find evidence of five distinct social participation trajectories between the ages of 35 and 71; the majority of which demonstrate social disengagement over time. Two, these participation declines are primarily attributable to changes in meeting friends and group exercise activity. Three, the most pronounced activity differences separating those in more favorable and unfavorable participation trajectories are cultural event attendance and voluntary group membership. Lastly, I identify particular high school activities that are associated with social participation decades later. In total, these results highlight heterogeneity among different types of social activities, and underscore the possible consequences of membership decisions made in early adulthood.

